# K-mer Content Changes with Node Degree in Promoter–Enhancer Network of Mouse ES Cells

**DOI:** 10.3390/ijms22158067

**Published:** 2021-07-28

**Authors:** Kinga Szyman, Bartek Wilczyński, Michał Dąbrowski

**Affiliations:** 1 Laboratory of Bioinformatics, Nencki Institute of Experimental Biology, 02-093 Warsaw, Poland; kinga@szyman.org.pl; 2 Faculty of Mathematics, Informatics and Mechanics, University of Warsaw, 02-097 Warsaw, Poland; bartek@mimuw.edu.pl

**Keywords:** Hi-C, 4-mer, dinucleotide, CpG, S/MAR, embryonic stem cell

## Abstract

Maps of Hi-C contacts between promoters and enhancers can be analyzed as networks, with cis-regulatory regions as nodes and their interactions as edges. We checked if in the published promoter–enhancer network of mouse embryonic stem (ES) cells the differences in the node type (promoter or enhancer) and the node degree (number of regions interacting with a given promoter or enhancer) are reflected by sequence composition or sequence similarity of the interacting nodes. We used counts of all k-mers (k = 4) to analyze the sequence composition and the Euclidean distance between the k-mer count vectors (k-mer distance) as the measure of sequence (dis)similarity. The results we obtained with 4-mers are interpretable in terms of dinucleotides. Promoters are GC-rich as compared to enhancers, which is known. Enhancers are enriched in scaffold/matrix attachment regions (S/MARs) patterns and depleted of CpGs. Furthermore, we show that promoters are more similar to their interacting enhancers than vice-versa. Most notably, in both promoters and enhancers, the GC content and the CpG count increase with the node degree. As a consequence, enhancers of higher node degree become more similar to promoters, whereas higher degree promoters become less similar to enhancers. We confirmed the key results also for human keratinocytes.

## 1. Introduction

It is well known that cis-regulatory regions of metazoans are split into promoters—proximal to transcription start sites (TSSs) and regions distal to TSSs, of which the most studied are enhancers, defined by ability to enhance the expression of genes by interaction with promoters, which typically involves looping out of the intervening chromosome segment [1,2]. Recently, with the establishment and refinement of chromosome conformation capture methods [3,4,5], it has become possible to study promoter–enhancer interactions by loop formation at a genome-wide scale. From such studies, it has become apparent that in the mouse embryonic stem (ES) cells, the majority of enhancers interact over relatively short distance with just one promoter, while promoters typically interact with several enhancers, and also there are many enhancers, in particular super-enhancers that interact with multiple promoters [4,6].

Promoters and enhancers and their interactions form a network, which can be regarded as a graph, with promoters and enhancers as nodes and interactions as edges. In such a graph, it is possible to ask questions about relationships between graph properties of nodes and edges and characteristics of the underlying regulatory regions and their interactions.

In the current study, we address a general question, whether the graph properties of nodes and edges in the promoter–enhancer interactions graph are reflected at the level of the genomic sequence of interacting regulatory regions. In particular, we have asked whether the type of the node (promoter vs. enhancer) and its node degree are associated with the sequence characteristics of the interacting promoters and enhancers.

We assessed the sequence composition and the sequence similarity of interacting regions through analysis of their DNA sequence k-mer content, for k = 4, with selected results confirmed for k = 1 and k = 2. The k-mer content was used before for identifying enhancers, for distinguishing enhancers from promoters and distinguishing among enhancers with different tissue specificity [7,8,9]. K-mers, or a related operation of sequence convolution, were also used for prediction of individual promoter–enhancer interactions [10,11,12].

Prior work related to the network node degree focused on single nucleotide composition of regulatory regions [13], while the effect of the CpG dinucleotide content was analyzed for related but distinct network parameters [14,15]. In particular, Lecellier et al. (2018) [13] demonstrated the effect of G or C (GC) content of enhancers on their node degree and the probability of localization at chromatin loop boundaries/anchors. However, a direct analysis of effects of sequence composition other than single nucleotide content of regulatory regions on their node degree has not been reported.

In this study, we analyzed the comprehensive data on promoter–enhancer interactions in the mouse embryonic stem (ES) cells recently published by Sahlén et al. (2015) [4]. We report that interacting promoters are GC-rich as compared to enhancers, in which all 4-mers containing CpGs are strongly under-represented relative to the remaining 4-mers. Promoters are more similar (have smaller k-mer distance) to their interacting enhancers than vice-versa. Most importantly, in both promoters and enhancers, the content of CpG-containing and of GC-rich 4-mers increases with the node degree. Largely as a consequence of that, and the long-known higher GC and CpG content of promoters than of enhancers [15,16], the k-mer distance of interacting nodes increases with the promoter degree and decreases with the enhancer degree, i.e., enhancers of higher node degree become more similar to promoters, whereas higher degree promoters become less similar to enhancers. We confirmed our key result that the CpG and GC content increase with the node degree also for another cell type/species, by analysis of the published data for the human keratinocytes [17]. The key novel aspects of the current work include correlation between the node degree and the CpG content for promoters, analysis the local similarity of the interacting regions, and over-representation of S/MARs patterns in enhancers.

## 2. Results

In the current study, we used the data on the mouse embryonic stem (ES) cells, comprising of 94,943 interactions between 15,905 promoters and 71,985 enhancers recently published by Sahlén et al. (2015) [4]. These data were obtained using the Hi-Cap method, which is a high-resolution Hi-C (average restriction fragment size under 1 kb), followed by sequence capture of interactions involving promoters’ regions.

The average length of enhancers was 699 bp, and of promoters, 585 bp. For straightforward comparison and interpretation of k-mer count vectors, we standardized the length of both types of regulatory regions at 1 kb, centered at the TSS or at the middle genomic position of the enhancer. Guided by our previous experience [8], we used k-mers of the length 4 because the shorter k-mers are less informative, while the longer are represented in too few data elements, given the data size. The steps of our sequence and graph analysis are illustrated in Figure S1.

### 2.1. Promoters Contain More GC-Rich k-mers, While Enhancers Have Reduced Content of All k-mers Containing CpG

**Figure 1:**
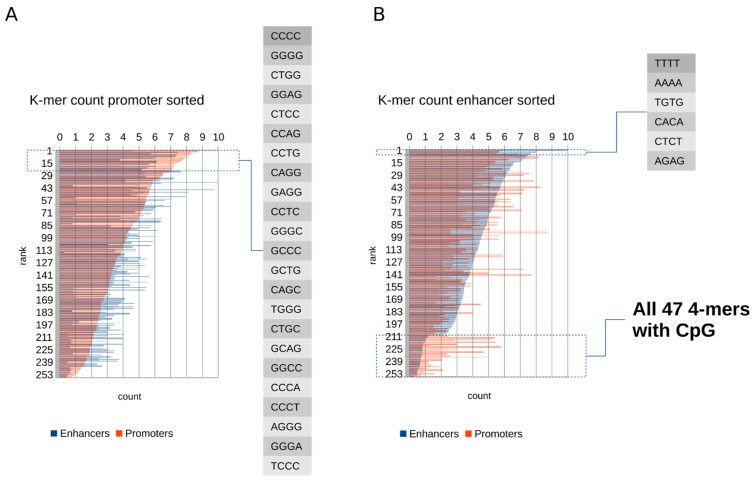
Ranked average k-mer (k = 4) counts in promoters and in enhancers. (**A**) Average k-mer counts in promoters and in enhancers ranked on their count in promoters. The dotted rectangle marks the top 23 k-mers with the highest counts in promoters that were also higher than their counts in enhancers. (**B**) Average k-mer counts in promoters and in enhancers ranked on their count in enhancers. The upper dotted rectangle marks the top six k-mers with the highest counts in enhancers that were also higher than their counts in promoters. The corresponding data with the k-mer sequences are provided as Table S1.

Analysis of the sequence k-mer (k = 4) content revealed that standardized 1 kb promoters and enhancers from the Sahlén’s dataset differ greatly in their average k-mer composition. A relatively smaller number of GC-rich 4-mers (with G or C at 4 or 3 positions) had higher counts in promoters than in enhancers, while a relatively larger number of AT-rich or AT/GC-balanced 4-mers had higher counts in enhancers than in promoters (Figure S2). Ranking all k-mers on their average count in promoters revealed that the ranked list of top 23 k-mers with the highest counts in promoters that were also higher than their counts in enhancers was headed by CCCC and its reverse complement GGGG, followed by 21 k-mers containing 4 or 3 Gs or Cs—notably none of them containing the CpG dinucleotide (Figure 1A). Ranking all k-mers on their average count in enhancers revealed a shorter ranked list of just 6 k-mers with the highest counts in enhancers that were simultaneously higher than their counts in promoters. This list was headed by TTTT followed by its reverse complement AAAA, but notably the remaining 4 k-mers most frequent in enhancers had balanced AT/GC content, with alternating W (IUPAC base symbol for T or A) and S (IUPAC G or S). Even more interestingly, the same ranking revealed that all 47 possible 4-mers containing the CpG dinucleotide are clearly under-represented and the least frequent among the 4-mers observed in standardized enhancer regions (Figure 1B and Table S1).

### 2.2. Content of All 4-mers Containing CpG and of GC-Rich 4-mers Increases with the Node Degree in Both Promoters and Enhancers

We were interested if k-mer content changes as a function of the node degree. To address this question, for each k-mer we computed a profile of its average count as the function of the node degree for degrees from 1 to 10. This analysis was performed separately for promoters and enhancers (as illustrated in Figure S1D).

**Figure 2:**
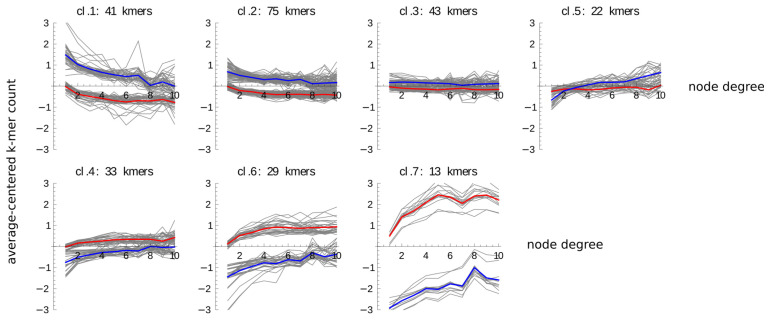
Clustered average k-mer count profiles of promoters and enhancers as the function of the node degree. The profiles of average counts of every k-mer as the function of the node degree were computed for degrees 1–10. This was done separately for promoters and for enhancers, and the two resulting profiles (vectors of length 10 indexed by the node degree) for the same k-mer in the promoters and in the enhancers were joined (head to tail) in that order, resulting in vectors of length 20. These vectors were then centered by subtraction of the joint profile average value and clustered using the k-medoid algorithm (Mathematica v. 8, Wolfram Research), with the number of statistically significant clusters determined automatically using the gap statistics. The results of the clustering were cut between the promoter and the enhancer part and the two halves are plotted alongside each other as the function of the node degree. The promoter part is marked by plotting its median profile in red and the enhancer part by plotting its median profile in blue.

We were particularly interested, how the profiles of the same k-mers compare between the promoters and the enhancers. To address this question, we joined each k-mer’s promoters and enhancers profile, resulting in a joint profile of length 20. Each joint profile was then centered by subtraction of its mean value and the resulting profiles for all the 256 k-mers were clustered. This clustering yielded seven clusters (Figure S3) with several interesting characteristics, which are better visible when the promoter and enhancer halves of the joint profiles are overlaid over each other (Figure 2). First and most important, five clusters, collectively grouping majority of k-mers, represented monotonous count changes with the node degree; with counts decreasing in two clusters (cl. 1, 2) and increasing in three clusters (cl. 4, 6, 7). In the remaining two clusters, the median counts were either not changing with the node degree (cl. 3), or changing only for the enhancers but not the promoters (cl. 5). Second, with the exception of cluster 5 there was a remarkable agreement between the patterns of change with the node degree between the promoters and the enhancers; however, the base levels for these changes were clearly different between the two groups of sequences. For clusters 1 and 2 of decreasing profiles, and also clusters 3 and 5, the counts were higher in enhancers, while for the clusters 4, 6, and 7 of increasing profiles the counts were higher in promoters, with bigger promoter–enhancer differences associated with larger count changes with the node degree.

**Figure 3:**
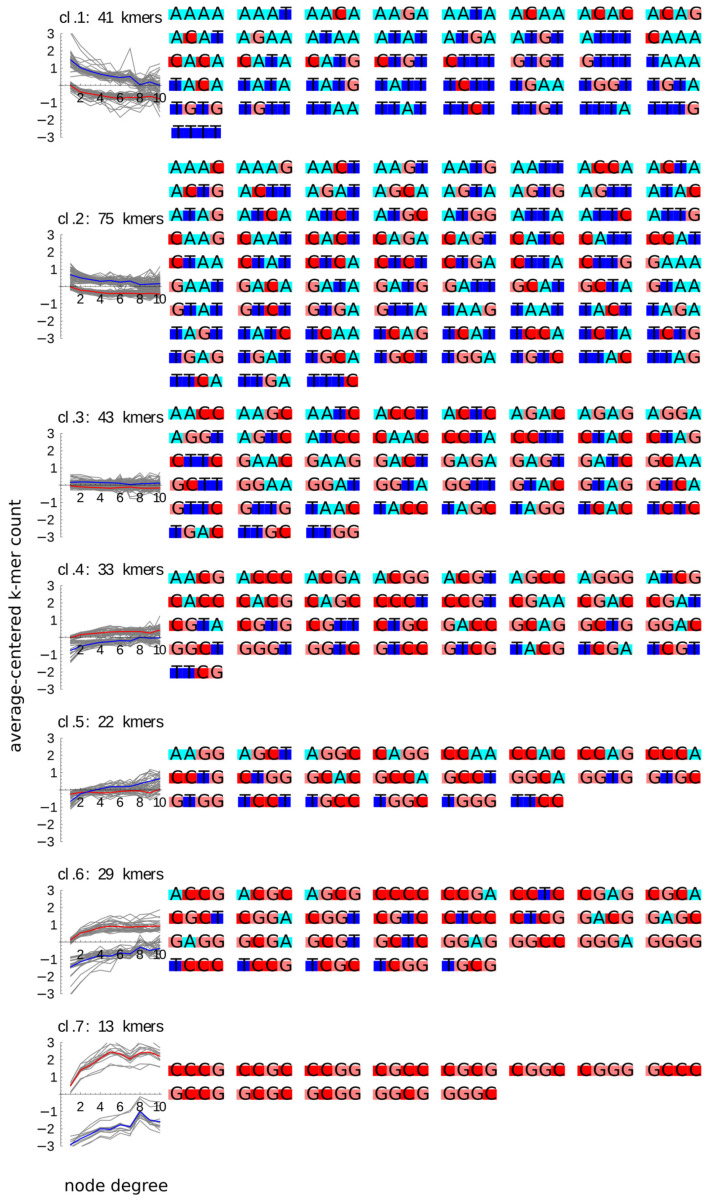
Base composition of the k-mer clusters (same as in Figure 2). The sequences of k-mers in each cluster are plotted alongside the profiles of this cluster, with every nucleotide marked by a different character background color: A-cyan, T-blue, G-pink, C-red.

To analyze an association between the sequences of k-mers and their average counts as the function of the type and degree of the containing node, we visualized the sequences of k-mers in every cluster. First, we inspected the AT vs. GC content of every k-mer after encoding them by colors (Figure 3). This inspection revealed that the content GC-rich k-mers was generally increasing with the node degree and higher in promoters than in enhancers. In particular, cluster 7, of the k-mers whose count increases most strongly with the node degree and with the highest promoter–enhancer difference, comprised only of 4-mers composed entirely of Gs or Cs—although not all of them, as three such GC-only 4-mers. None of them with the CpG dinucleotide were present in cluster 6 grouping profiles with more moderate increases with the node degree. At the other end of the GC content, AT-only k-mers were most numerous in clusters 1 and 2, of profiles decreasing with the node degree and higher counts in enhancers.

The situation for 4-mers composed of three G or C was more interesting; while majority of them were in clusters 4 and 6 of the profiles increasing with the node degree, as many as 16 of such k-mers were found in cluster 5 of profiles showing enhancer-specific increases with the node degree—notably, none of them with the CpG dinucleotide.

The situation for 4-mers with equal AT and GC content was the least clear-cut. Although they formed an overwhelming majority (41 of 43 members) of cluster 3 of profiles not changing with the node degree, they also constituted 27 members of cluster 2 of small decreases with the node degree—none of them with the CpG dinucleotide, 10 members of cluster 4 of small increases with the node degree—all of them with the CpG dinucleotide, and 5 of 22 members of cluster 5 of enhancer-specific increases—none of them with the CpG dinucleotide.

**Figure 4:**
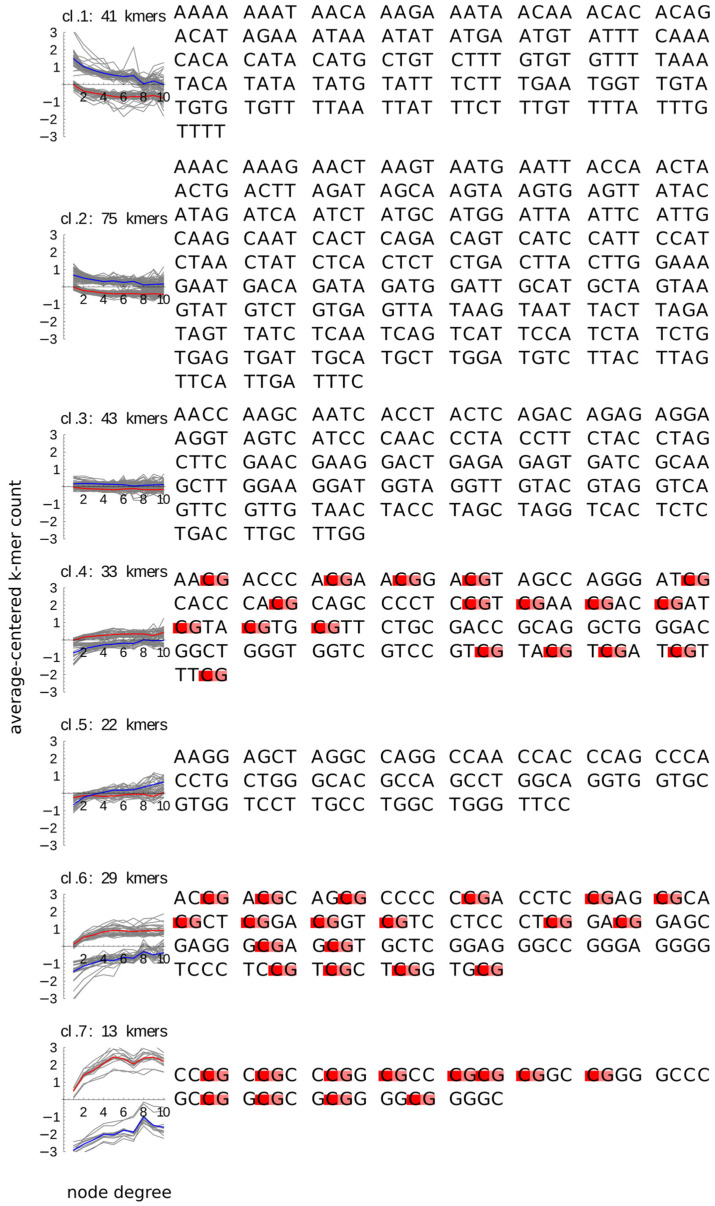
CpG content of the k-mer clusters (same as in Figure 2). The sequences of k-mers in each cluster are plotted alongside the profiles of this cluster, with CpG dinucleotides (in the 5′-3′ orientation) marked by colors: G-pink, C-red.

We checked if the content of CpG pairs discriminated between different patterns of k-mer count changes with the node degree and type (Figure 4). We found out that presence of (one or more) CpG dinucleotides distinguishes perfectly the k-mers whose count increases with the node degree both in promoters and in enhancers (clusters 4, 6, 7) from all the remaining k-mers including the k-mers whose count decreases with the node degree (clusters 1, 2), does not change with the node degree (cluster 3), or increases with node degree but only in enhancers and not in promoters (cluster 5).

### 2.3. Promoters Are More Similar to Their Interacting Enhancers Than Vice-Versa

Above, we have shown that promoters have higher average content than enhancers of GC-rich 4-mers. We have also demonstrated that the content of all GC-rich 4-mers increases with the node degree. This is true for both promoters and enhancers, which, together with their different average GC-content, causes enhancers of higher degree to become more similar to promoters in their k-mer content. The above analysis was global—performed over all promoters and enhancers. Below we address the same question locally, for pairs of interacting promoters and enhancers. As the measure of sequence dissimilarity, we used the Euclidean distance between the k-mer count vectors of an interacting promoter and enhancer pair, referred to as their k-mer distance (illustrated in Figure S1E). We were interested in a local measure of dissimilarity distinguishing between promoters and enhancers. To obtain such a measure, for each promoter we computed the average of its k-mer distances to all the enhancers it interacts with, and conversely, for each enhancer we computed the average k-mer distance to all the promoters it interacts with. In graph terms, for each node we computed its average k-mer distance over all the promoter–enhancer edges of this node. We call the result the local average k-mer distance (lakd, illustrated in Figure S1F). We then analyzed the distribution and the average values of this measure (i.e., of lakd) over all nodes of a particular type and/or degree.

**Figure 5:**
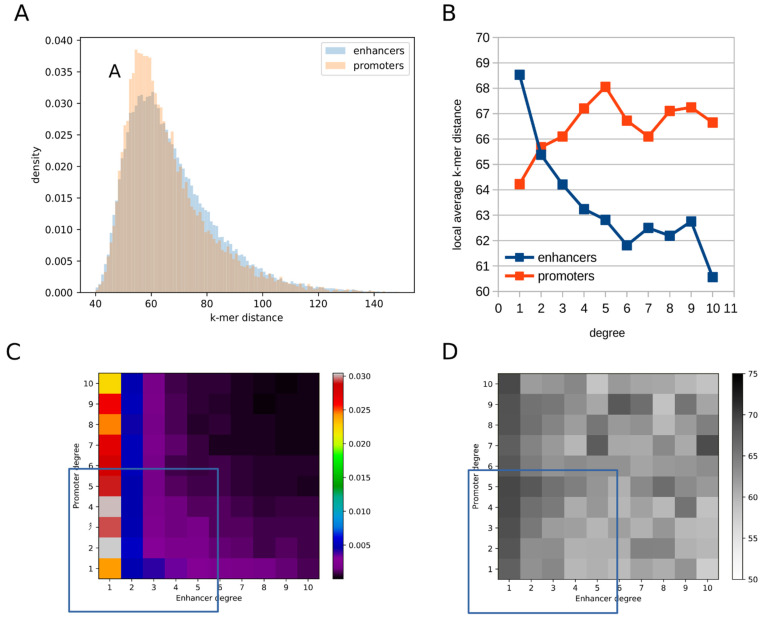
The average k-mer distance of promoters and of enhancers to the nodes they interact with (the local average k-mer distance—lakd), in the promoter–enhancer network of mouse ES cells. (**A**) Distributions (density) of the local average k-mer distance (marked “k-mer distance”) in all promoters and all enhancers. (**B**) Average values of the lakd of promoters and of enhancers as the function of their node degree. (**C**) Two-dimensional frequency distribution of all the promoter–enhancer interactions (edges) over their promoter and enhancer node degrees, with the frequency (fraction of the interactions with a given promoter degree and enhancer node degree) represented by the color scale. (**D**) Average k-mer distance along the edges (represented by the gray level) as the function of their promoter and enhancer node degree.

The local average k-mer distance is not symmetrical with regard to grouping the interactions by the common promoter or, alternatively, by the common enhancer. The distributions of this measure for promoter–enhancer interactions after grouping them by promoters and by enhancers are shown in Figure 5A. The distribution for the promoters is shifted towards smaller k-mer distances, as compared to the distribution for the enhancers. This difference between the two distributions is highly statistically significant (Kolmogorov–Smirnov test *p*-value 2.6 × 10^−47^), indicating that the local similarity of the promoters to the enhancers they interact with is greater than the local similarity of the enhancers to the promoters they interact with.

### 2.4. Higher-Degree Enhancers Are More Similar to the Promoters They Interact with, While the Reverse Is True for Promoters

When the distributions of the local average k-mer distance after grouping by enhancers or, alternatively, by promoters, were stratified by the (promoter–enhancer) node degree, we observed that changes in the node degree are associated with changes in the average (over all nodes of a given type and degree) of the local average k-mer distance (Figure 5B). Notably, the directions of these changes were opposite for grouping by the promoters and for grouping by the enhancers. For grouping by the enhancers, the local average k-mer distance decreases with the enhancer degree (linear regression slope −0.93, *p*-value 2.4 × 10^−65^; Spearman correlation −0.06, *p*-value 1.7 × 10^−60^), whereas for grouping by the promoters the local average k-mer distance increases with the promoter degree (linear regression slope 0.08 *p*-value 3.5 × 10^−8^; Spearman correlation 0.13, *p*-value 3.8 × 10^−70^). Thus, enhancers of higher degree become more similar to promoters in their k-mer content not only globally but also locally—for the interacting promoter–enhancer pairs. The opposite is true for the promoters, which become on average less similar to their interacting enhancers with the increasing promoter degree. The above changes of the k-mer distance with the node degree are monotonic between the degrees 1 and 5 of both promoters and enhancers (Figure 5B). This region (marked by the blue square in Figure 5C) covers most of the promoter–enhancer interactions, as can be seen from their two-dimensional frequency distribution over the promoter degree and the enhancer degree (Figure 5C).

We were interested, if the opposite effects of the promoter node degree and of the enhancer node degree on the promoter–enhancer sequence similarity are due to the same or different sets of promoter–enhancer interactions. To investigate this, we grouped the interactions (network edges) simultaneously by their promoter degree and the enhancer degree. We focused on the region between the degrees 1 and 5 of both promoters and enhancers, covering most of the interactions. For the edges in this region, the average k-mer distance (represented by the gray level in Figure 5D) decreases with the enhancer degree, whereas it increases with the promoter degree. We conclude that the same interactions contribute to the opposite effects of the promoter node degree and of the enhancer node degree on the promoter–enhancer sequence similarity.

### 2.5. GC and CpG Content Increases with the Node Degree in Mouse ES Cells and Also in Human Keratinocytes

**Figure 6:**
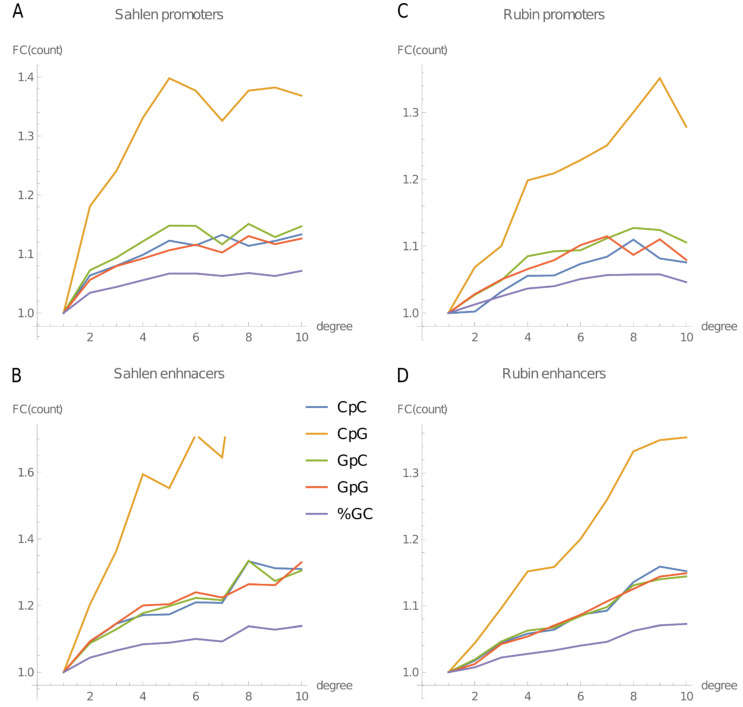
Relative changes of the average GC content (%GC) and of the average counts of the four dinucleotides of G or C in promoters and enhancers as the function of their node degree. FC—the fold change relative to the degree 1. (**A**,**B**) Data from the mouse ES cells. (**C**,**D**) Data from the human keratinocytes. (**A**,**C**) Promoters. (**B**,**D**) Enhancers.

The changes of the 4-mers’ counts with the node degree in the promoter–enhancer network of mouse ES cells are clearly related to the GC content and the CpG count of the 4-mers, suggesting that changes in the k-mers count with the node degree largely reflect underlying changes of the GC content and the counts of dinucleotides of G or C in the regulatory regions. We therefore directly checked how these parameters of the regulatory regions change with node degree. The analysis was performed separately for promoters and for enhancers. We found out that for both promoters and enhancers, the average GC content and the average counts of every possible dinucleotide of G or C increase with the node degree (Figure 6A,B). The relative changes (fold change) of the CpG counts with the increase in the node degree were steeper than the relative changes of the counts of the remaining (G or C)-dinucleotides, and these in turn were steeper than the relative changes of the GC content (% GC). The above observations were true for the mouse ES cells (Sahlén’s dataset, Figure 6A,B), and we confirmed them also for an additional dataset of promoter–enhancer interactions in human keratinocytes (Figure 6C,D), obtained using the promoter capture Hi-C (CHi-C) performed during epidermal differentiation, published by Rubin et al. (2017) [17].

The correlations between the GC content or (G or C)-dinucleotide counts and node degree are all positive and highly significant (Table 1), confirming that the relationship between the GC content and the node degree holds not only for the mouse ES cells but also for differentiated cells (keratinocytes) in human. The differences in the counts of the four dinucleotides of G or C with the node increasing from 1 to 10 degree were all similar (Figure S4), but CpGs had the lowest counts of the four (Figure S5), resulting in the highest relative change.

## 3. Discussion

We analyzed graph properties of promoter–enhancer interactions network of mouse embryonic ES cells published by Sahlén et al. (2015) [4] augmented with the results of our k-mer (k = 4) analysis of the genomic sequence of the interacting promoters and enhancers. We found that on average promoters contain more instances of GC-rich and CpG-containing 4-mers than enhancers. The fact that promoters have high GC content and often harbor CpG islands (CGIs) is well known [16], and these sequence characteristics are important for establishing nucleosome-free regions [18,19]. We note, however, that none of the top 23 k-mers with the highest counts in promoters (all of them GC-rich and with higher counts in promoters than in enhancers) contained a CpG dinucleotide. We found that in enhancers all possible CpG-containing 4-mers are strongly underrepresented, as compared to the remaining 4-mers, in agreement with previous findings of Andersson et al. (2014) [15] that FANTOM enhancers are depleted of CGI.

Another interesting and novel observation is that the top six k-mers with the highest counts in enhancers, which correspond to the top three double-stranded k-mers, map—in the same order—to the three most abundant mono- and dinucleotide repeat patterns ([A]n, [AC]n, [AG]n) observed in human scaffold/matrix attachment regions (S/MARs), with S/MARs defined as the union of ChIP-Seq regions for 14 proteins that bind to S/MARs [20].

Most importantly, we demonstrated for the first time that the content of all possible 4-mers containing one or more CpG dinucleotide (also those with a balanced AT vs. GC content) and of GC-rich k-mers increases with the promoter–enhancer node degree. We confirmed and statistically validated the dependency between the node degree and the GC or CpG content of regulatory regions by a direct calculation of the GC percentage and of the counts of dinucleotides of G or C in the regulatory regions. We moreover checked that these findings are valid also for another species and a differentiated cell type (human keratinocytes).

Of the two datasets compared in our current study, the promoter–enhancer interactions reported by Rubin et al. (2017) [17] were filtered by a requirement that the distal region (nonbait fragment) overlaps a ChIP-seq peak of H3K27Ac—a mark of active enhancers, whereas such a filtering step was not performed during the generation of the dataset of Sahlén et al. (2015) [4], which contained all non-bait regions, of which 64% had chromatin marks of enhancers. In a more recent work, Sahlén et al. (2021) [21] showed a positive correlation between the node degree and the H3K27Ac peak overlap, in the system studied by Rubin et al. (2017) [17], i.e., human keratinocytes undergoing differentiation in vitro. In the current work, the proportionality between the node degree of enhancers and their CpG and GC content was observed for both compared datasets—i.e., irrespective of the H3K27Ac filtering step, suggesting that the effect of the sequence (CpG) content on the enhancer node degree is not mediated by histone acetylation. In this context, we note that both Rubin et al. (2017) [17] and Sahlén et al. (2021) [21] point to the importance of pre-established promoter–enhancer contacts during keratinocytes differentiation. We speculate that the sequence (CpG) content could shape in particular these contacts.

The datasets of Sahlén et al. (2015) [4] and of Rubin et al. (2017) [17] used in the current study were produced using different restriction enzymes (4-cutter MboI vs. 6-cutter HindIII), which could have hampered the comparison due to very different fragment length distributions. There are several reasons for which this did not happen. First, the ranges and the distributions of the node degrees of both promoters and enhancers although not identical are similar between the two datasets, meaning that that longer loops detected following HindIII digestions compensate for the shorter loops that could only be observed following MboI digestion. In that sense, the two choices of the restriction enzyme can be regarded as complementary—probing loops of different length. Second, the effects of the node type (promoter, enhancer) and of the node degree on the CpG and GC content were analyzed separately within either dataset. Third, before analysis of the sequence content of the interacting regions we standardized both their length and the genomic location in the same way for the two node types and for the two datasets, namely for the bait region (promoter) we choose a 1 kb fragment centered at the gene start (TSS), and for the nonbait region (enhancer) we also choose a 1 kb fragment, this time centered at the center of the nonbait fragment in the respective dataset. The 1 kb size is similar to the average size of the bait (885 bp) and nonbait fragments (699 bp) in the dataset of Sahlén et al. (2015) [4] and also to the typical size of the segments of nonbait fragments that overlap H3K27Ac peaks in the study of Rubin et al. (2017) [17]. The uniform and biologically sound fragment length permitted meaningful comparison of the k-mer frequencies between promoters and enhancers and also between the two datasets.

Our current results extend to promoters the results of Lecellier et al. (2018) [13], who demonstrated that enhancers with higher (above the median) GC content contact more promoters and have higher probability of localization at chromatin loop boundaries/anchors. Our work also complements the work of Gu et al. (2016) [14], who documented a monotonic dependence between chromatin interaction (CI) frequency of a pair of Hi-C bins and their average GC and CpG content in human ES cells and IMR90 fibroblasts [14]. We note that the two measures (CI and the node degree), although related, are distinct. CI measures the interaction frequency between a pair of genomic loci, while the node degree measures the number genomic loci interacting with a given locus (Figure S6A vs. Figure S6B).

The sequence analysis of interacting promoters and enhancers was performed on unmasked genome because we did not want to exclude repetitive elements [22] many of which contribute to cis-regulatory regions [23]. The choice of using the unmasked genome was crucial for the observation that the CpG content increases the node degree because, as shown by Gu et al. (2016) [14], it is the SINE Alu elements that contribute the high CpG and GC content of the interacting genomic loci.

In a related work focused on promoters Bajic et al. (2006) [24] classified promoters based on their GC content upstream and downstream of the TSS and found that majority of promoters have high GC content in both segments. Therefore, from our current results, it can be predicted that a majority of promoters have high node degrees, which agrees well the observed values [4,17].

The dependence between the 4-mer count and the node degree was strongest for the 4-mers with high GC content, which also had the largest average count differences between promoters and enhancers. Notably, however, presence of even one CpG marked the 4-mer for an increased count with the increased node degree (Figure 4). Moreover, the relative changes of the CpG dinucleotide count with the node degree are steeper than of the other dinucleotides of G or C, which in turn are steeper than the changes of the (single nucleotide) GC content. The above findings suggest that the key element of the GC content that is associated with the node degree are the CpGs. This conclusion is in agreement with the observations of Andersson et al. (2014) [15] that ubiquitous enhancers are more likely to overlap both the CGI and the sites of cohesin binding [15]. While the above observations of Andersson et al. (2014) [15] and the results of Lecellier et al. (2018) [13] mentioned above suggested a possible link between the CpGs and the node degree, it is our work that shows that this dependency exists. The CpG counts behave in the same way in promoters and enhancers, suggesting that the same CpG-dependent mechanism operates for both types of regulatory regions. We speculate that one such mechanism could be methylation of histone H3K9me1 to H3K9me3 directed by CpG-binding proteins [25,26,27] followed by compartmentalization/phase separation.

In addition to the GC-rich and/or CpG-containing 4-mers that increased their count with the node degree of both promoters and enhancers, we also observed a smaller set of 4-mers that increased their count with the node degree specifically for enhancers but not promoters. We note that many of them contained GG or CC dinucleotides.

By analysis of the k-mer distance between interacting promoter–enhancer pairs, we also unexpectedly found that promoters are on average more similar to their interacting enhancers than enhancers to their interacting promoters. We speculate that enhancers, which are typically interacting with a single promoter [4], have more freedom to match the sequence of this promoter, whereas the promoters, typically interacting with multiple enhancers [4], have less freedom to adapt to each of them. Indeed, an extensive overlap of transcription factor binding site motifs in promoters have been described [28].

Because promoters have higher average content of GC-rich k-mers and of CpGs than enhancers, the observed changes in their content with the node degree mean that enhancers of higher node degree become more similar to promoters, whereas promoters of higher node degree become less similar to enhancers (Figure S6C). Andersson et al. (2014) [15] reported that motifs identified de novo in enhancers resemble more those identified in the non-CGI promoters then in the CGI promoters [15]. The results we obtained using the k-mer distance agree with these findings and additionally show a dependence of the promoter–enhancer sequence similarity on the node degree. Our results add to the recent findings that CGI-associated enhancers bear promoter-like characteristics [29], which in our case is the higher node degree. The similarities of high-degree enhancers to promoters are consistent with the hypothesis that the structure and function of enhancers, in particular of higher node degrees, and promoters can be very similar [30,31].

## 4. Materials and Methods

### 4.1. Data

We used the data on 94,943 interactions between 15,905 promoters and 71,985 enhancers published by Sahlén et al. (2015) [4] as additional information (Additional file 1; Supplementary Table 5v5.xlsx), which we downloaded from the journal website: https://genomebiology.biomedcentral.com/articles/10.1186/s13059-015-0727-9 (accessed on 10 January 2018).

We standardized the length of both types of regulatory regions at 1 kb, centered at the TSS or at the middle genomic position of the enhancer. The sequence data of the standardized regulatory regions were taken from the mm9 assembly version of the mouse genome. The promoter–enhancer interaction data of Rubin et al. (2017) [17] contained in the file: GSE84660_ChiC_contacts_b2g.bed can be found under this link: https://www.ncbi.nlm.nih.gov/geo/query/acc.cgi?acc=GSE84660 (accessed on 10 January 2018).

### 4.2. K-mer Count

We used Jellyfish [32] to count instances of all 256 possible 4-mers in the +1 strand of the genomic sequence of every standardized regulatory region, resulting in k-mer counts (k-mer count vectors or k-mer vectors for short) for every regulatory region (Figure S1A,B). The Jellyfish software can be found under this link: http://www.genome.umd.edu/jellyfish.html (accessed on 10 January 2018). We annotated each sequence with its type (promoter or enhancer) and the node degree (number of promoter–enhancer interactions). For every 4-mer we computed their average count over the nodes of a particular type (promoters, enhancers) (Figure S1C).

We were interested if k-mer content changes as a function of the degree of the node. To address this question, for each k-mer we computed a profile of its average count as the function of the node degree for degrees from 1 to 10. This analysis was performed separately for promoters and enhancers (Figure S1D). We were particularly interested in how the profiles of the same k-mers compare between the promoters and the enhancers. To address this question, we joined each k-mer’s promoters and enhancers profile, resulting in a joint profile of length 20. To facilitate interpretation of 256 such profiles, we centered each joint profile by subtraction of its average value and then clustered the centered joint profiles. For the clustering we used the k-medoid algorithm (Mathematica v. 8, Wolfram Research), with the number k (here indicating the number of statistically significant clusters, not the k-mer length) automatically determined with the gap statistics [33].

### 4.3. Local Average k-mer Distance

The Euclidean distance between two k-mer count vectors (of length 256 for k = 4), corresponding to two regulatory regions (a promoter and an enhancer), named the k-mer distance, was used as a measure of sequence dissimilarity between these regions (Figure S1E). For each promoter we then computed the average of its k-mer distances to all the enhancers it interacts with, and conversely, for each enhancer we computed the average k-mer distance to all the promoters it interacts with. In graph terms, for each node we computed its average k-mer distance over all the promoter–enhancer edges of this node. We call the result the local average k-mer distance (lakd, Figure S1F). We then analyzed the distribution and the average values of this measure (i.e., of lakd) over all nodes of a particular type and/or degree.

### 4.4. Code Availability

Our Python code used in this study can be found under this link: https://github.com/kinga322/kmers (accessed on 10 January 2018).

## Figures and Tables

**Table 1:** Correlation of node degree with the GC content and the counts of (G or C) dinucleotides.

Dataset and Type	Correlation	*p*-Value	Slope
Sahlén promoters			
GG	0.1580	2.28 × 10^−89^	0.3456
CC	0.1621	4.9 × 10^−94^	0.3702
GC	0.1824	4.68 × 10^−119^	0.3497
CG	0.2073	6.51 × 10^−154^	0.5035
%GC	0.1934	7.08 × 10^−134^	0.1285
Sahlén enhancers			
GG	0.1392	2.29 × 10^−308^	2.1587
CC	0.1337	3.62 × 10^−284^	2.1534
GC	0.1579	0	1.6845
CG	0.1345	1.27 × 10^−287^	1.1236
%GC	0.1696	0	0.7159
Rubin promoters			
GG	0.0743	4.66 × 10^−25^	0.1355
CC	0.0722	9.51 × 10^−24^	0.1490
GC	0.0957	1.66 × 10^−40^	0.1970
CG	0.1203	3.28 × 10^−63^	0.2834
%GC	0.0851	2.15 × 10^−32^	0.0649
Rubin enhancers			
GG	0.1118	1.54 × 10^−157^	0.9189
CC	0.1107	1.55 × 10^−154^	0.8581
GC	0.1292	6.36 × 10^−210^	0.6348
CG	0.1309	1.36 × 10^−215^	0.3858
%GC	0.1292	3.68 × 10^−210^	0.3121

## Data Availability

We used the data on interactions between promoters and enhancers published by Sahlén et al. (2015) [4] as additional information (Additional file 1; Supplementary Table 5v5.xlsx), which we downloaded from the journal website: https://genomebiology.biomedcentral.com/articles/10.1186/s13059-015-0727-9 (accessed on 10 January 2018). The promoter–enhancer interaction data of Rubin et al. (2017) [17] contained in the file: GSE84660_ChiC_contacts_b2g.bed can be found under this link: https://www.ncbi.nlm.nih.gov/geo/query/acc.cgi?acc=GSE84660 (accessed on 10 January 2018).

## Supplementary Materials

The following are available online at https://www.mdpi.com/article/10.3390/ijms22158067/s1, Legends to Figures S1–S6: Legends to the supplementary figures, Figure S1: Schematic illustration of the sequence and graph analysis, Figure S2: Scatter plot of average k-mer counts in promoters and in enhancers, Figure S3: Clustered joint k-mer count profiles as the function of the node degree, Figure S4: Differences of the average GC content (%GC) and of the average counts of the four dinucleotides of G or C in promoters and enhancers grouped by the node degree, Figure S5: The average GC content (%GC) and of the average counts of the four dinucleotides of G or C in promoters and enhancers grouped by the node degree, Figure S6: Graphical summary of the results, Table S1: Average k-mer counts in promoters and in enhancers.
