# Supplementary material for: K-mer Content Changes with Node Degree in Promoter–Enhancer Network of Mouse ES Cells
Source: Int J Mol Sci. 2021 Jul 28;22(15):8067. doi: 10.3390/ijms22158067 (PMC8347099; doi:10.3390/ijms22158067)
Supplement: Supplementary file 1 [file ijms-22-08067-s001.zip › ijms-1259806_rev1 Supplementary Materials/Legends to figures S1-S6.pdf]

Legends to figures S1-S6

Figure S1. Schematic illustration of the sequence and graph analysis.

(**A**) The k-mer content of a sequence (k=4). (**B**) Illustration of the k-mer count vector (alias the k-mer vector or the k-mer counts) of a sequence (k=4). (**C**) Illustration of the average k-mer counts of promoters and enhancers. (**D**) Illustration of a k-mer count profile – the average k-mer counts of a particular k-mer in promoters or enhancers grouped by their node degree. (**E**) The k-mer distance of the k-mer vectors of two sequences. (**F**) Local average k-mer distance. In the promoter-enhancer network graph, the nodes represent promoters (red) or enhancers (blue) and the edges (orange) – their interactions. With each edge we associate as its weight the k-mer distance between the nodes at its ends. The integer weights (shown as numbers and also as the edges' widths) serve to illustrate by example the computation of lakd, as the sum of the weights of the edges of each node divided by its degree.

Figure S2. Scatter plot of average k-mer counts in promoters and in enhancers.

For greater clarity (and in this figure only) the typically very similar (data not shown) average counts of complementary k-mers were added. This was done separately for promoters and for enhancers, and only one (alphabetically earlier) k-mer from every complementary pair was plotted at xy-position corresponding to the values of these sums in promoters and in enhancers.

Figure S3. Clustered joint k-mer count profiles as the function of the node degree.

The profiles of average counts of every k-mer as the function of the node degree were computed for degrees 1-10. This was done separately for promoters and for enhancers, and the two resulting profiles (vectors of length 10 indexed by the node degree) for the same k-mer in the promoters and in the enhancers were joined (head to tail) in that order, resulting in vectors of length 20. These vectors were then centered by subtraction of the joint profile average value and clustered using the k-medoid algorithm (Mathematica v. 8, Wolfram Research), with the number of statistically significant clusters determined automatically using the gap statistics.

Figure S4. Differences of the average GC content (%GC) and of the average counts of the four dinucleotides of G or C in promoters and enhancers as the function of their node degree.

Δ – difference relative to the degree 1. (**A**, **B**) Data from the mouse ES cells. (**C**, **D**) Data from the human keratinocytes. (**A**, **C**) Promoters. (**B**, **D**) Enhancers.

Figure S5. The average GC content (%GC) and of the average counts of the four dinucleotides of G or C in promoters and enhancers as the function of their node degree.
(**A**, **B**) Data from the mouse ES cells. (**C**, **D**) Data from the human keratinocytes. (**A**, **C**) Promoters. (**B**, **D**) Enhancers.

Figure S6. Graphical summary of the results.
(**A**, **B**) Illustration of the difference between (**A**) the chromatin interaction (CI) frequency used in the study of Gu et al. (2016) [14], represented by intensity of the red color, and (**B**) the node degree used in the current study. For simplicity, CIs are shown only in the upper-diagonal halves of the simplified Hi-C contact matrices. (**C**) A schematic illustration how the changes in GC and CpG content make the sequences of higher degree enhancers more similar to those of the promoters.
