# Supplementary figures and images for: K-mer Content Changes with Node Degree in Promoter–Enhancer Network of Mouse ES Cells
Source: Int J Mol Sci. 2021 Jul 28;22(15):8067. doi: 10.3390/ijms22158067 (PMC8347099; doi:10.3390/ijms22158067)

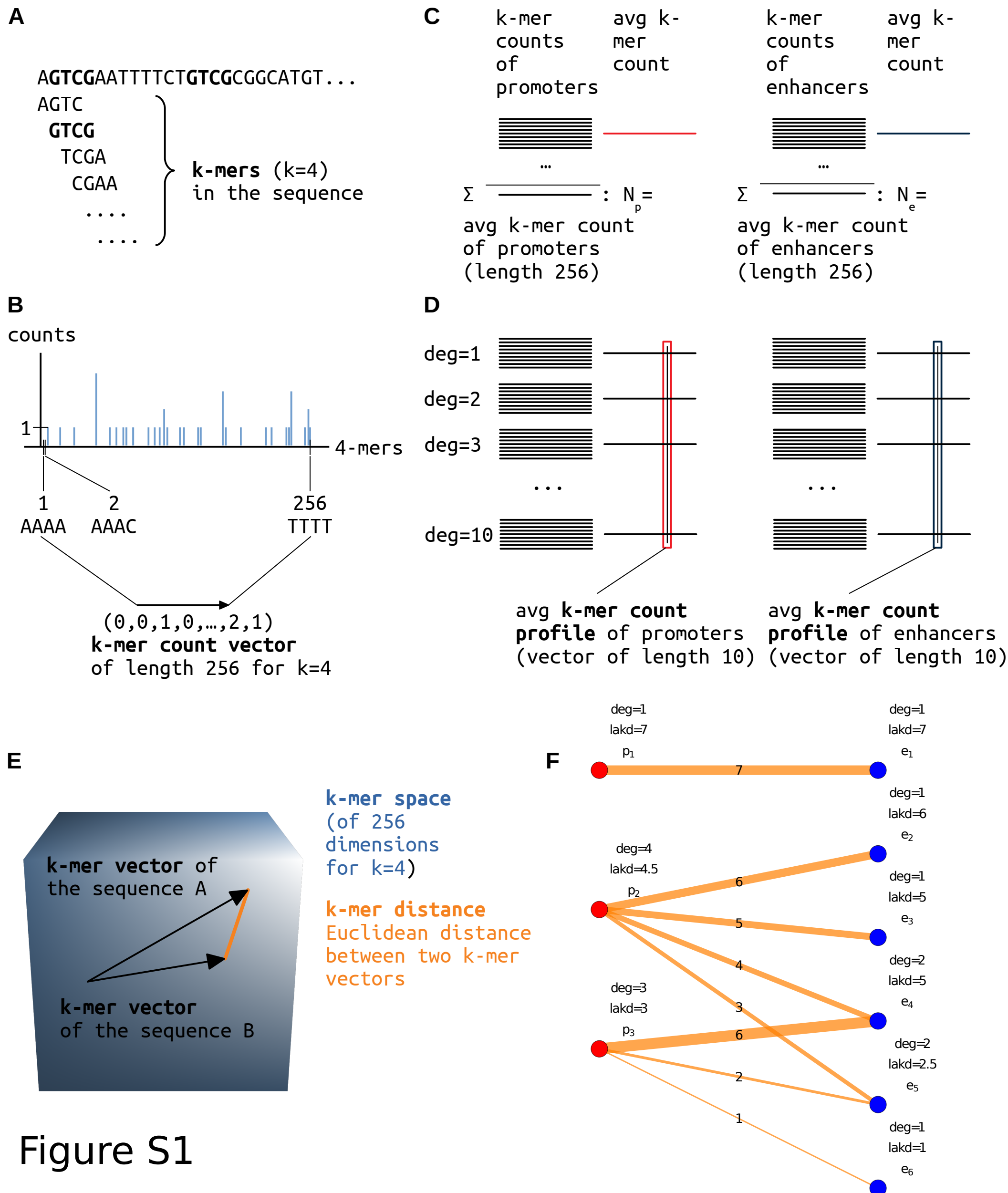

Figure S1

Supplement: Supplementary file 1 [file ijms-22-08067-s001.zip › ijms-1259806_rev1 Supplementary Materials/Figure_S1.pdf]

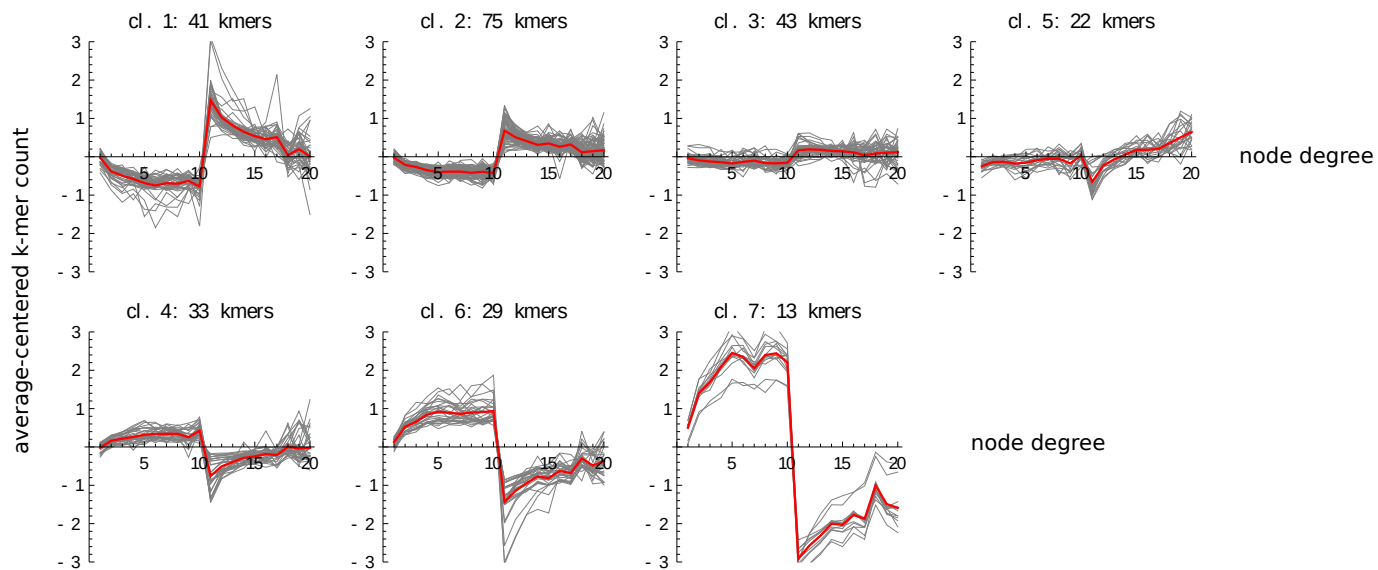

Figure S3

Supplement: Supplementary file 1 [file ijms-22-08067-s001.zip › ijms-1259806_rev1 Supplementary Materials/Figure_S3.pdf]

A

Sahlen promoters

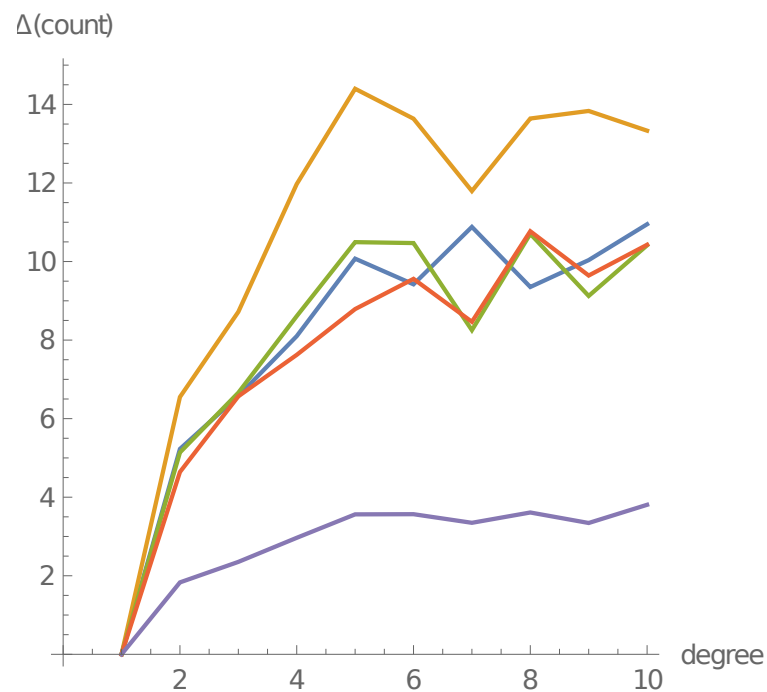

C

Rubin promoters

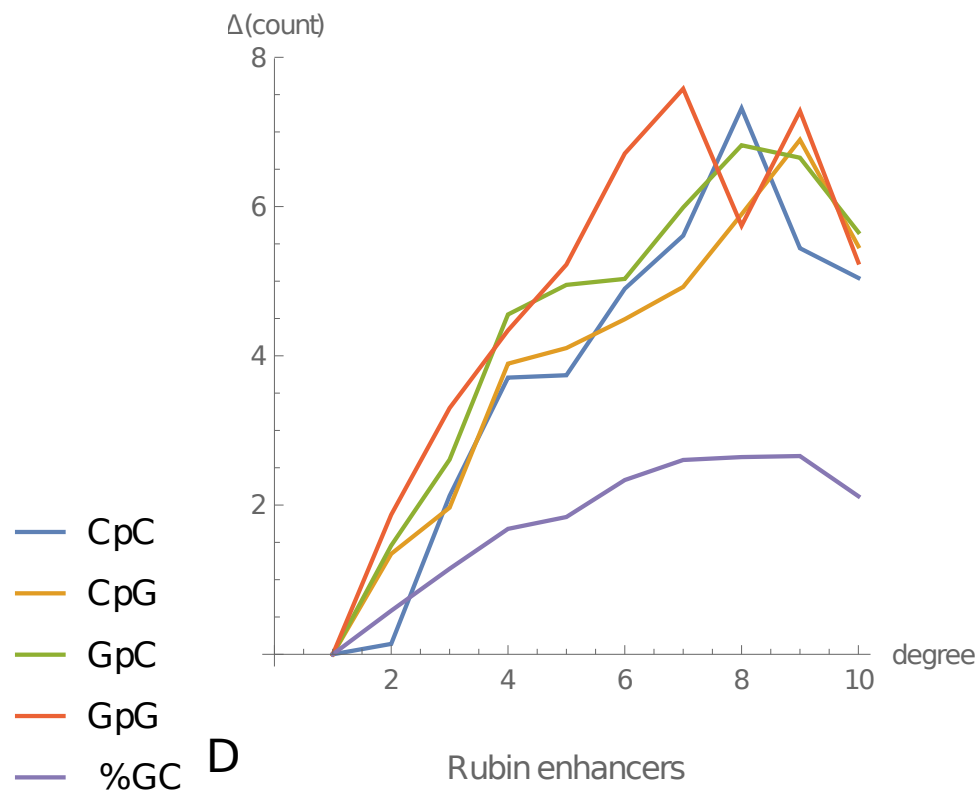

B

Sahlen enhancers

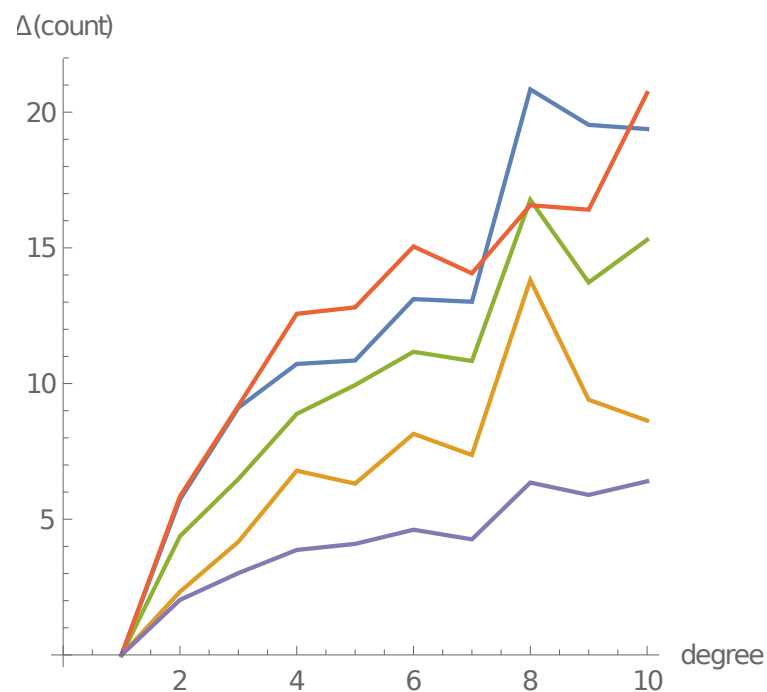

D

Rubin enhancers

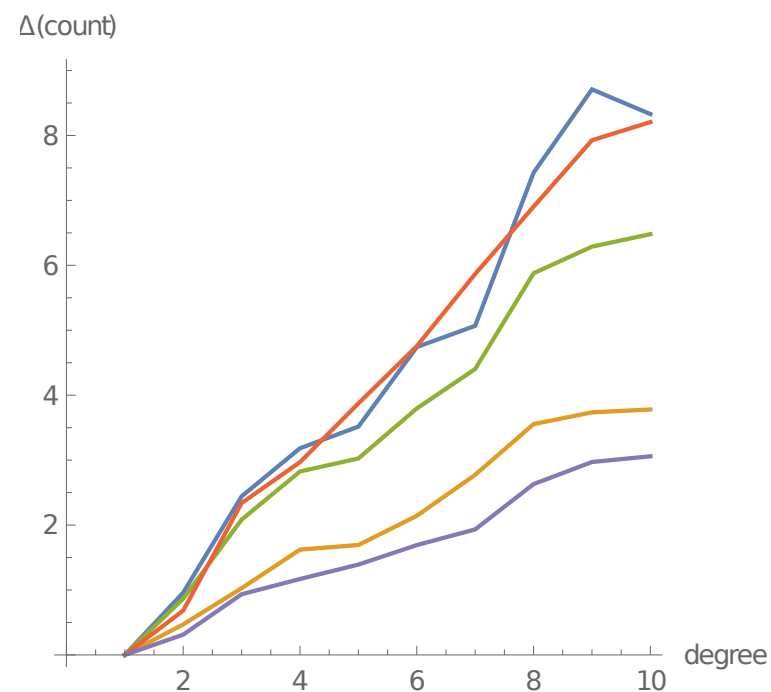

Figure S4

Supplement: Supplementary file 1 [file ijms-22-08067-s001.zip › ijms-1259806_rev1 Supplementary Materials/Figure_S4.pdf]

A

Sahlen promoters

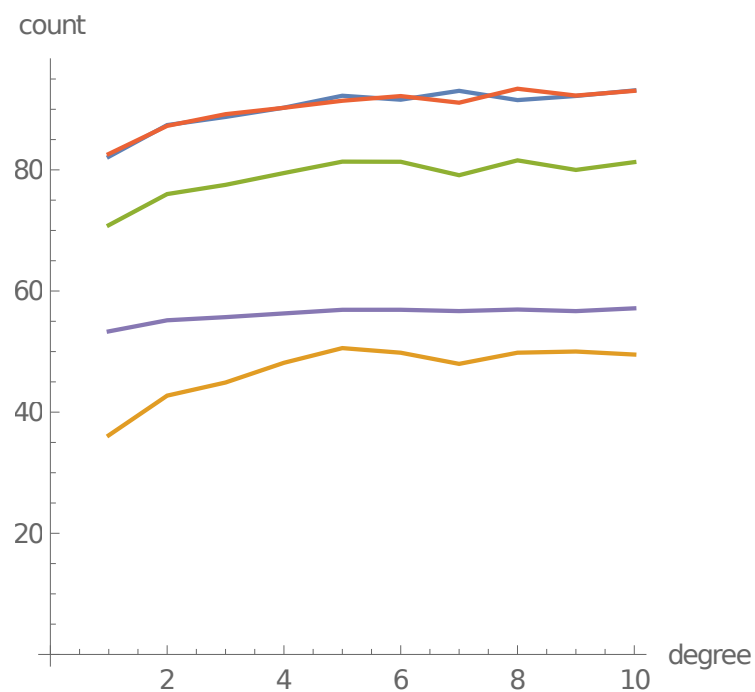

C

Rubin promoters

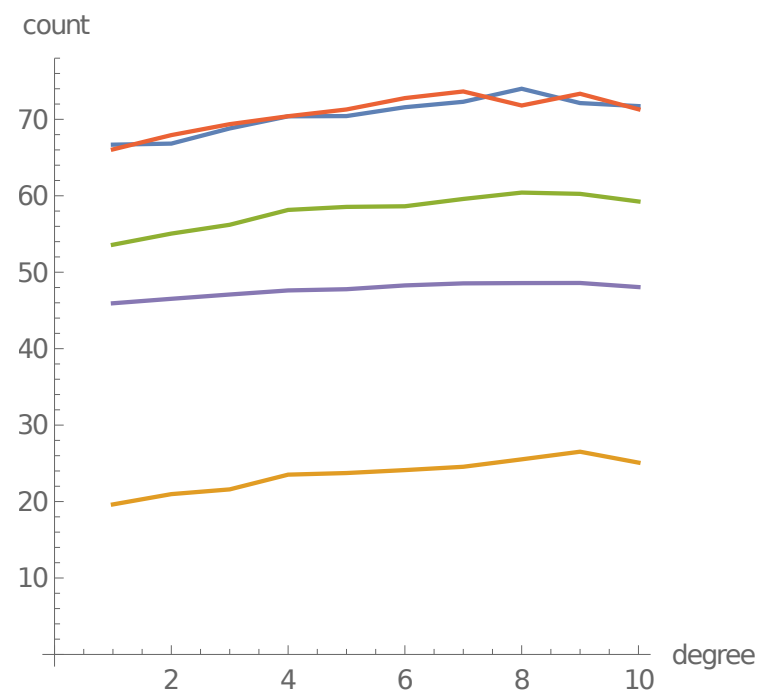

B

Sahlen enhancers

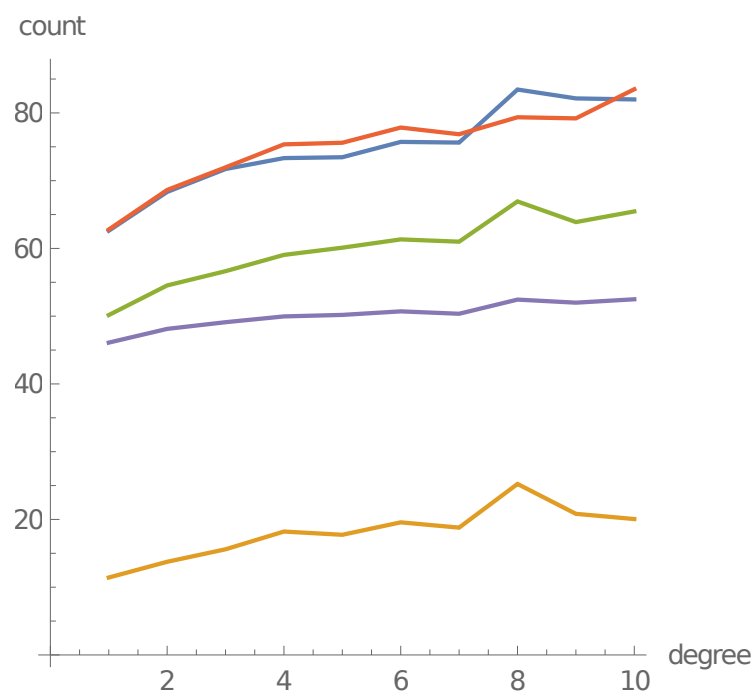

D

Rubin enhancers

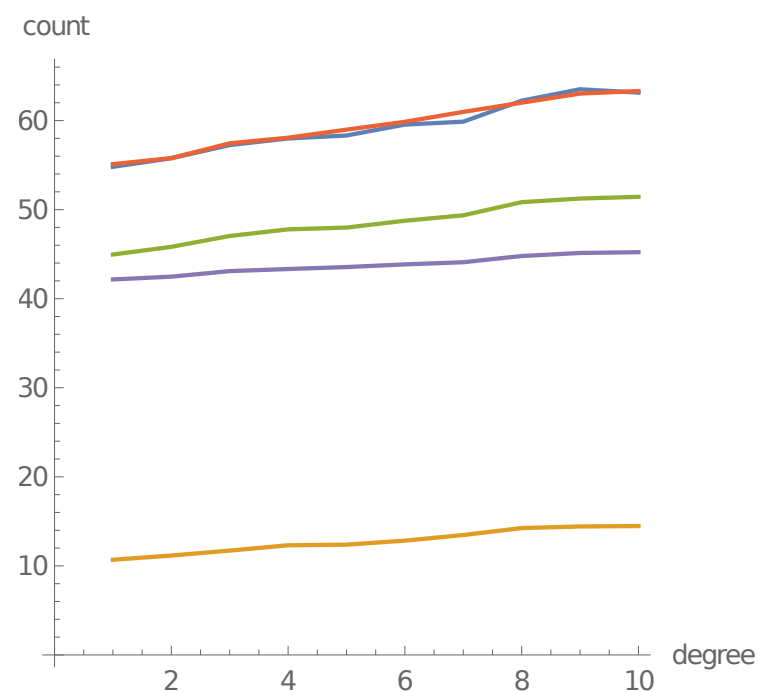

Figure S5

Supplement: Supplementary file 1 [file ijms-22-08067-s001.zip › ijms-1259806_rev1 Supplementary Materials/Figure_S5.pdf]

A

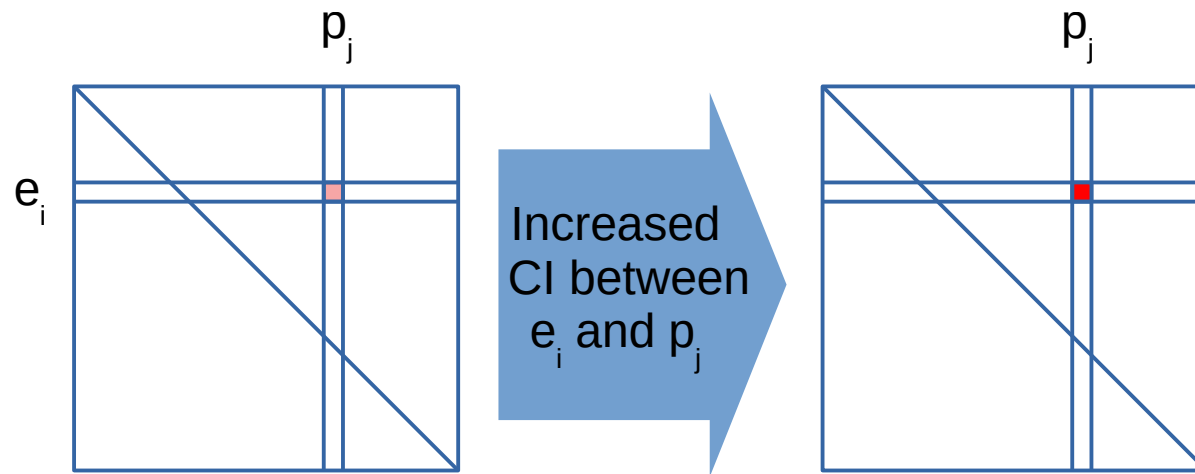

B

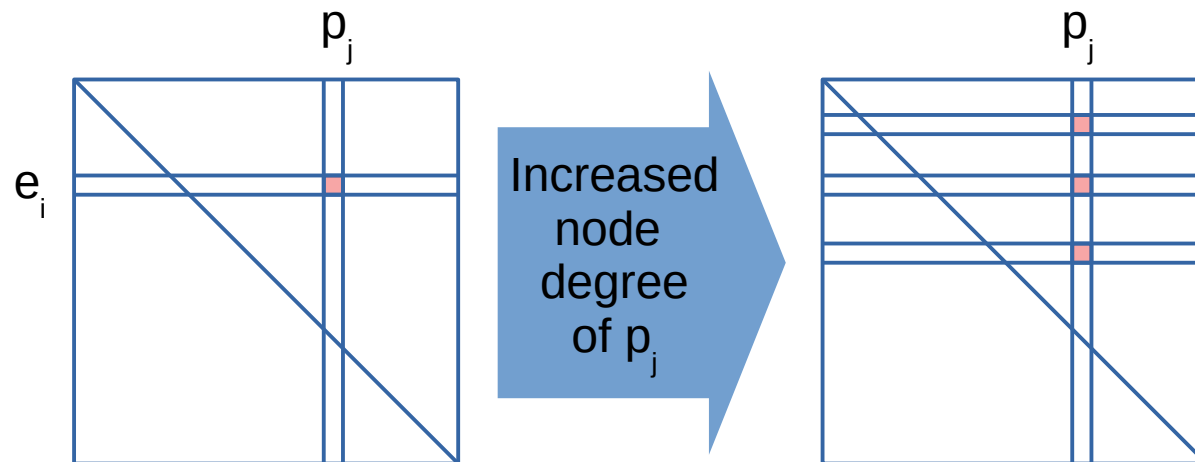

C

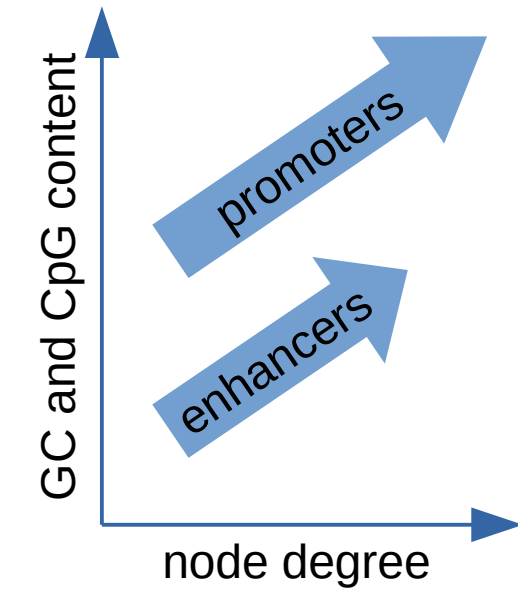

Figure S6

Supplement: Supplementary file 1 [file ijms-22-08067-s001.zip › ijms-1259806_rev1 Supplementary Materials/Figure_S6.pdf]
