# Supplementary material for: K-mer Content Changes with Node Degree in Promoter–Enhancer Network of Mouse ES Cells
Source: Int J Mol Sci. 2021 Jul 28;22(15):8067. doi: 10.3390/ijms22158067 (PMC8347099; doi:10.3390/ijms22158067)
Supplement: Supplementary file 1 [file Figure_S2.pdf › ijms-1259806_rev1 Supplementary Materials/Figure_S2.pdf]

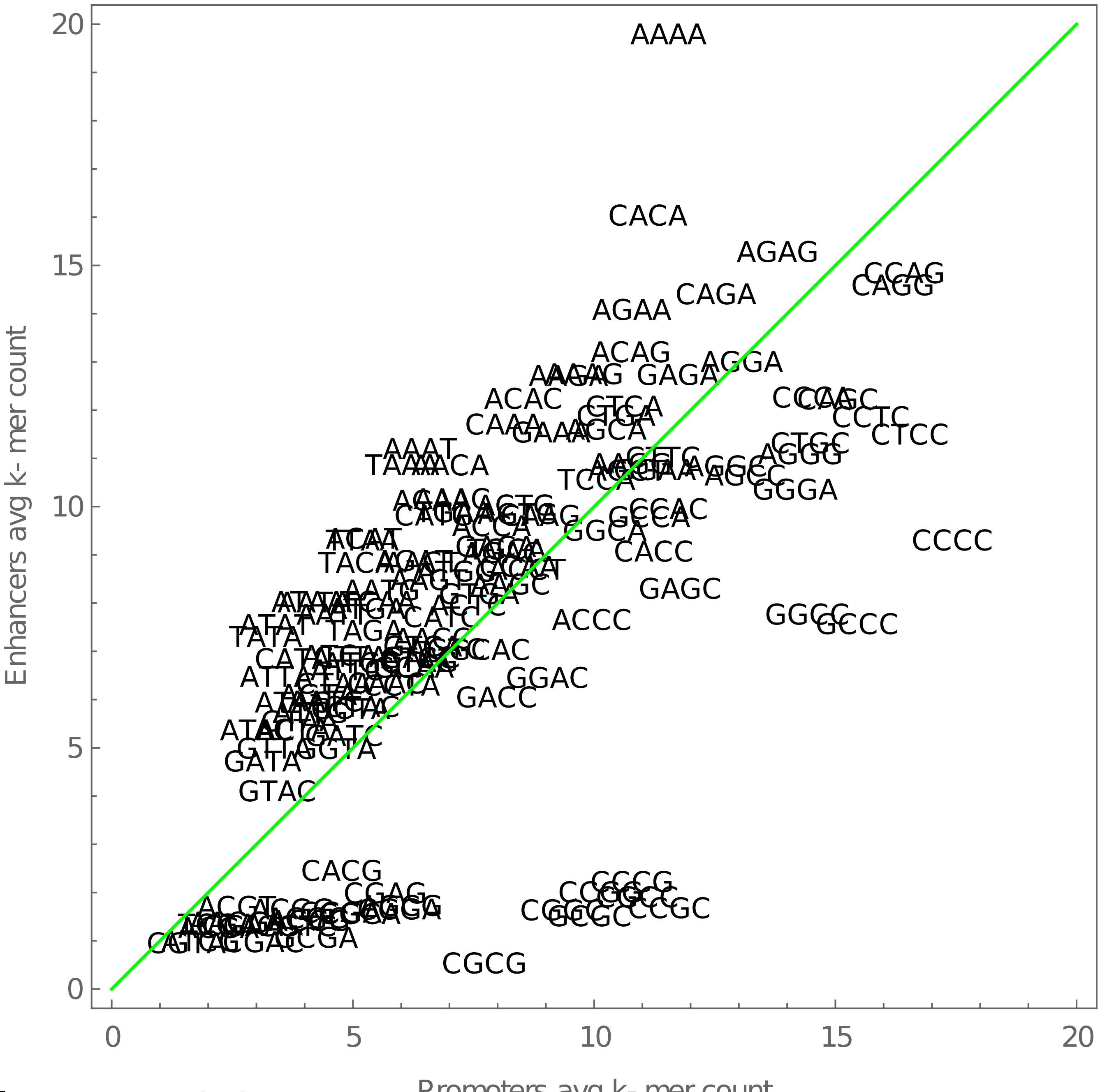
Enhancers avg k- mer count
Promoters avg k- mer count
0
5
10
15
20
0
5
10
15
20
AAAA
CACA
AGAG
CCAG
CAGG
CAGA
AGAA
ACAG
AGGA
GAGA
ACAC
CTCA
CTGA
CAAA
AGCA
CTCC
CCTC
CTGC
AGGG
GGGA
AGCC
TCCA
CCAC
GCCA
GGCA
CACC
CCCC
GAGC
GGCC
GCCC
ACCC
GGAC
GACC
TATA
GATA
GTAC
CACG
CGAG
CCCG
CCGC
CGGC
GCGC
CGCG
CGGA

Figure S2
